# Application Advances of Gold Nanoparticles in Cancer Theranostics: From Physicochemical Mechanisms to Multifunctional Nanoplatforms

**DOI:** 10.3390/ijms27083454

**Published:** 2026-04-12

**Authors:** Chunhui Wu, Maolin Qiao, Haiyang Ning, Tinging Gao, Huijuan Xu, Dengfeng Xue, Xinzheng Li

**Affiliations:** The Second Clinical Medical College, Shanxi Medical University, Taiyuan 030001, China; buyicongran@sxmu.edu.cn (C.W.); 17835154673@163.com (M.Q.); 202400220315@sxmu.edu.cn (H.N.); gaotingting@sxmu.edu.cn (T.G.); xuhuijuan0308@163.com (H.X.)

**Keywords:** gold nanoparticles, cancer theranostics, localized surface plasmon resonance, photothermal and photodynamic therapy, multifunctional nanoplatforms

## Abstract

The high morbidity and mortality of cancer pose a severe challenge to human health. Traditional diagnostic and therapeutic strategies still exhibit obvious limitations in early diagnostic sensitivity, therapeutic precision, and real-time monitoring of treatment efficacy. The development of nanotechnology has provided novel solutions for precision cancer theranostics. Among nanomaterials, gold nanoparticles (AuNPs) have become a research hotspot in tumor nanomedicine due to their tunable size and morphology, excellent localized surface plasmon resonance (LSPR) effect, and favorable biocompatibility. However, despite encouraging preclinical outcomes, several challenges hinder their clinical translation, including an incomplete understanding of long-term toxicity, complex in vivo biological interactions, the lack of standardized evaluation protocols, and regulatory uncertainties and manufacturing reproducibility issues. This paper systematically reviews the physicochemical and biological mechanisms of AuNPs in cancer theranostics, and summarizes the latest research advances of AuNPs in cancer detection and diagnosis (including biomarker detection and multimodal imaging) as well as in therapeutic fields, covering photothermal therapy (PTT), photodynamic therapy (PDT), radiosensitization, targeted drug and nucleic acid delivery, and immunotherapy-assisted strategies. Furthermore, we discuss the development of intelligent and stimuli-responsive theranostic nanoplatforms based on AuNPs, and outline their future prospects in precision medicine and personalized cancer therapy, with particular emphasis on the requirements for clinical translation, including safety evaluation, large-scale production, and regulatory approval pathways.

## 1. Introduction

Cancer represents a major disease associated with high incidence and mortality rates globally, and constitutes a serious threat to human health [1]. Although considerable progress has been made in therapeutic strategies, including surgery, radiotherapy, chemotherapy, and targeted therapy in recent years, traditional diagnostic and therapeutic paradigms still present substantial limitations [2]. Specifically, insufficient sensitivity for early diagnosis, restricted resolution of imaging techniques, the absence of real-time monitoring of therapeutic efficacy during treatment, and systemic toxicity arising from the non-specific distribution of chemotherapeutic agents collectively impede the further advancement of precision cancer medicine [3,4,5]. In addition, the high heterogeneity of tumors and the complexity of the tumor microenvironment (TME) further increase therapeutic challenges, meaning that monotherapeutic modalities frequently fail to achieve satisfactory therapeutic outcomes [6]. Accordingly, the development of integrated technological platforms combining high-sensitivity diagnostic capacity and precise therapeutic function has emerged as a key research direction in contemporary oncology.

The advent of nanotechnology has offered novel strategies for cancer diagnosis and therapy [7]. Nanoparticles possess unique physicochemical properties, including controllable size, large specific surface area, and facile surface functionalization, which enable them to interact specifically with biological systems at molecular and cellular levels [8,9]. Upon surface modification with targeting moieties such as antibodies, aptamers, or peptides, nanoparticles can achieve active targeted recognition and accumulation in tumor tissues [10]. Meanwhile, their outstanding drug-loading capacity and controllable release profiles contribute to elevated local drug concentrations at tumor sites and reduced damage to normal tissues [11]. Furthermore, certain nanomaterials exhibit distinctive optical, electrical, or magnetic properties, which can be exploited for imaging enhancement and synergistic therapy, thereby promoting the development of the theranostic concept [12,13].

Among various nanomaterials, inorganic metal nanoparticles have attracted considerable attention due to their stable structures and distinctive physicochemical properties [14]. Among these, gold nanoparticles (AuNPs) demonstrate remarkable advantages in tumor molecular imaging, drug delivery, photothermal therapy (PTT), and radiosensitization, owing to their excellent biocompatibility, outstanding localized surface plasmon resonance (LSPR) effect, and facile surface modification [15,16]. AuNPs with different morphologies, including nanospheres, nanorods, and nanoshells, exhibit tunable optical responses in the near-infrared (NIR) region, providing an essential foundation for deep-tissue imaging and PTT [17,18]. Accordingly, multifunctional theranostic platforms constructed on the basis of AuNPs have emerged as a prominent research focus in contemporary tumor nanomedicine.

However, despite these promising advances, several critical challenges remain, including the complexity of in vivo biological interactions, potential long-term toxicity, and the lack of standardized evaluation and regulatory frameworks, which collectively hinder their clinical translation. Therefore, a comprehensive and systematic understanding of the design principles, functional mechanisms, and translational barriers of AuNP-based systems is urgently needed.

In this review, we summarize the physicochemical and biological mechanisms of AuNPs in cancer theranostics and discuss recent advances in their applications in cancer detection, diagnosis, and therapy. In addition, we highlight emerging intelligent and stimuli-responsive nanoplatforms and address key challenges related to clinical translation and regulatory considerations, aiming to provide insights into the future development of AuNP-based precision cancer therapies.

## 2. Physicochemical and Biological Mechanisms of AuNPs in Cancer Theranostics

### 2.1. Physical Properties

**Figure 1:**
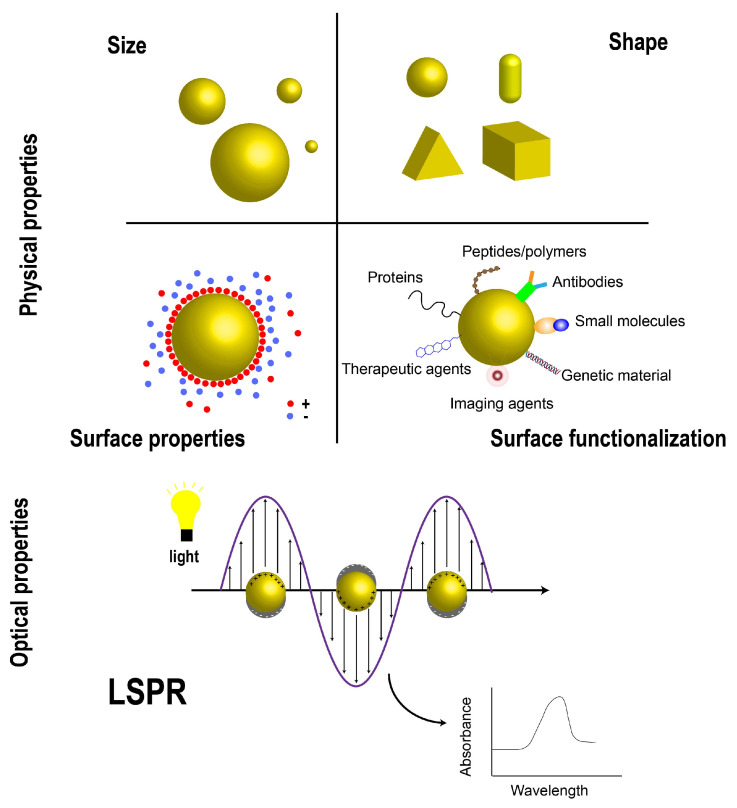
Schematic illustration of the size-, shape-, and surface-dependent physical properties of gold nanoparticles (AuNPs) and their localized surface plasmon resonance (LSPR)-mediated optical behavior. In the charge panel, “+” denotes positive charge and “−” denotes negative charge.

AuNPs, as representative noble metal nanomaterials, exhibit physical properties that are strongly dependent on the precise modulation of their size, shape, and interfacial structure (Figure 1). AuNPs are generally defined within the size range of 1–100 nm, and their size-dependent effects endow the materials with optical, electrical, and thermal behaviors distinct from those of bulk gold [18]. Variations in size not only influence the peak position and bandwidth of the LSPR but also significantly alter the specific surface area and surface energy, thereby regulating the stability and interfacial reaction kinetics of the nanoparticles [19,20,21]. Smaller AuNPs generally display a higher absorption contribution and stronger surface energy [22,23], whereas larger particles exhibit an enhanced scattering component, which is favorable for optical imaging applications [24].

Particle shape represents another critical parameter governing the physical behavior of AuNPs. AuNPs can be fabricated into diverse geometric morphologies, including spheres, rods, stars, and cubes, and shape differences lead to distinct plasmon resonance behaviors and localized electric field distributions [25,26]. These differences directly affect their functional performance in biomedicine [27]. For instance, spherical AuNPs are frequently utilized in drug delivery and imaging owing to their facile synthesis and readily modifiable surfaces [28]; gold nanorods and star-shaped nanoparticles, with their anisotropic optical properties, are more appropriate for PTT and photoacoustic imaging (PAI) [29,30]. To intuitively compare the key properties and representative applications of AuNPs with different morphologies, we have compiled Table 1, which summarizes their differences in size, physicochemical advantages, and major therapeutic and imaging applications, thereby highlighting the central role of morphology in determining their biomedical functions.

The surface properties of AuNPs further govern their behavior in complex biological environments. The surface charge of nanoparticles is commonly characterized by zeta potential, which not only influences the dispersion and stability of colloids but also directly modulates the interactions between particles, surrounding media, and biological membranes [51,52,53,54]. AuNPs with different surface charges exhibit remarkable differences in cellular uptake and mechanisms of membrane interaction, and such effects display complex interdependent behavior with particle size [55,56,57]. Accordingly, precise control over surface charge enables the modulation of biodistribution and cellular uptake pathways of nanoparticles.

Surface functionalization constitutes a vital strategy for achieving efficient and stable performance of AuNPs in practical applications. Owing to their surface chemical affinity, AuNPs can be functionalized with ligands, polymers, and biomolecules to improve their dispersibility, biocompatibility, and specific targeting capability [58,59]. Functionalization also modifies the local dielectric environment at the particle surface, thereby influencing the optical response and plasmon resonance properties [60]. Surface modification strategies not only enhance the stability of AuNPs in biomedical applications but also provide a design basis for their use as diagnostic imaging agents and therapeutic carriers [61,62]. Numerous studies have demonstrated that modification of AuNPs with various ligands (e.g., polyethylene glycol (PEG), antibodies, peptides, etc.) can significantly prolong their blood circulation time and improve targeted accumulation efficiency in vivo [63,64].

In summary, the physical properties of AuNPs are determined by the combined effects of size- and shape-dependent behaviors and interfacial chemistry. Precise engineering of particle size, shape, and surface properties—including surface charge and functionalization—enables the systematic modulation of the optical, electrical, and biointerfacial behaviors of AuNPs. This provides a fundamental physical basis as well as design principles for their applications in sensing, bioimaging, and targeted therapy.

### 2.2. Optical Properties

AuNPs are characterized most distinctively by their unique and tunable optical properties, whose fundamental physical origin lies in the LSPR effect (Figure 1). When exposed to incident electromagnetic radiation, the conduction band free electrons of AuNPs undergo collective oscillations, giving rise to prominent absorption and scattering peaks at specific wavelengths [65,66]. Mechanistically, this resonance process originates from the collective oscillation of free electrons in metallic nanostructures driven by electromagnetic fields. When the frequency of incident light matches that of electron oscillation, resonant absorption occurs and substantially enhances the local electromagnetic field intensity. Subsequently, the excited electrons rapidly reach thermal equilibrium via electron–electron relaxation, and transfer energy to lattice vibrations through electron–phonon coupling. Finally, the energy is released into the surrounding environment as heat through phonon–phonon relaxation. This non-radiative decay pathway constitutes the essential physical basis for the photothermal effect of AuNPs [67].

Such plasmonic resonance behavior is strongly dependent on particle size, morphology, and the dielectric constant of the surrounding medium, thus enabling precise modulation of their optical responses through nanostructure engineering [68,69]. For example, as particle size decreases, the scattering probability of electrons on the nanoparticle surface increases and the plasmon damping effect is enhanced, resulting in a blue shift in the LSPR peak and an increased optical absorption component [70,71]; in contrast, as particle size increases, radiative decay and light scattering effects are gradually enhanced, rendering larger AuNPs more advantageous for applications in optical imaging and scattering enhancement [72].

In particular, anisotropic structures (e.g., gold nanorods, nanoshells, and nanostars) allow tunable red-shifting of the resonance peak into the NIR region (700–1100 nm) [73]. Compared with spherical particles, anisotropic structures can generate multiple plasmon oscillation modes. For instance, gold nanorods possess both transverse and longitudinal resonance modes, among which the longitudinal mode corresponds to a longer electron oscillation path, thereby lowering the plasmon oscillation frequency and shifting the resonance peak toward longer wavelengths [74,75,76]. By tuning the aspect ratio of nanorods or the shell thickness of nanoshells, precise modulation of the LSPR peak position can be achieved to shift it into the NIR biological window [33,77]. Meanwhile, particles with tip structures such as nanostars can generate strong local electromagnetic field “hotspots” at the tips or nanogaps, significantly boosting light absorption efficiency and improving photothermal conversion performance [78,79].

As the NIR window exhibits low absorption and scattering in biological tissues, referred to as the “biological transparency window”, this spectral tunability provides a crucial physical basis for deep-tissue tumor imaging and PTT [80,81]. Furthermore, the surface chemical properties of AuNPs also influence their LSPR behavior. When nanoparticle surfaces are modified with ligands, polymers, or biomolecules, the local dielectric environment and refractive index surrounding the particles are altered, resulting in a shift in the plasmon resonance peak [82]. According to the classical Mie scattering theory, the LSPR frequency is closely related to the electron density of the metal and the dielectric constant of the surrounding medium. Thus, the formation of a surface molecular layer can modulate the optical response by changing the local refractive index environment [83,84]. Such synergistic regulation of size, shape, and surface chemistry enables AuNPs to achieve systematic optimization of light absorption, local electromagnetic field enhancement, and photothermal conversion efficiency at the nanoscale.

**Figure 2:**
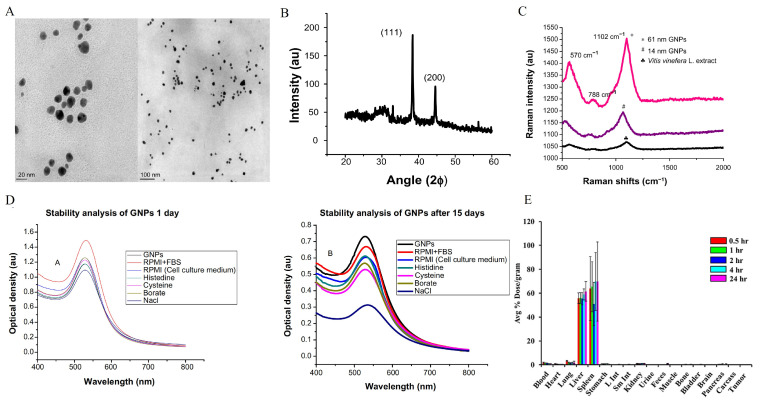
(**A**) Transmission electron microscopy (TEM) images showing the size and morphology of gold nanoparticles (GNPs). (**B**) X-ray diffraction (XRD) patterns of GNPs. (**C**) Raman spectra from GNPs of different sizes: black, grape extract; blue, spectrum from 14 ± 1 nm nanoparticles; pink, spectrum from 61 ± 2 nm particles. (**D**) Stability and cytotoxicity of GNPs. UV-vis spectra of the GNPs in various buffers at various time periods: (A) 1 day; (B) 15 days. Reproduced from Ref. [88] under the terms of the Creative Commons license. (**E**) Biodistribution of RESV–^198^AuNPs in mice following intravenous injection, expressed as %ID g^−1^. Reproduced from Ref. [89] under the terms of the Creative Commons license. AuNPs, gold nanoparticles.

The strong localized electromagnetic field generated by LSPR serves as the key mechanism underlying surface-enhanced Raman scattering (SERS). The plasmon-induced electromagnetic field significantly amplifies the Raman scattering signal at the “hotspots” of nanoparticles, improving detection sensitivity by several orders of magnitude and, thus, enabling ultrasensitive molecular detection of tumor-related biomarkers [85,86]. Upon further modification of gold nanoparticle surfaces with targeting molecules such as antibodies or aptamers, selective recognition and quantitative analysis of cancer-specific molecular markers can be achieved, providing technical support for early molecular diagnosis and dynamic monitoring of tumors [87]. Kalmodia et al. [88] developed a green synthesis method using Vitis vinifera extract to produce noncytotoxic Raman-active AuNPs, which were characterized by transmission electron microscopy (TEM), X-ray diffraction (XRD), and Raman spectroscopy (Figure 2A–C).

In terms of therapeutic applications, the PTT effect of AuNPs originates from the non-radiative decay of plasmons. Upon NIR light irradiation, the absorbed photon energy is rapidly converted into localized thermal energy via electron–phonon and phonon–phonon relaxation processes, leading to a rapid temperature increase in the tumor region [90]. This hyperthermic effect induces protein denaturation, destruction of the cytoskeletal structure, and mitochondrial dysfunction [91]. Compared with conventional hyperthermia, AuNPs-based photothermal therapy exhibits advantages such as precise spatial localization and minimal damage to surrounding normal tissues [92].

In addition to the direct photothermal effect, AuNPs can also enhance the therapeutic efficacy of photodynamic therapy (PDT). Although gold itself is not a classical photosensitizer, its plasmon-enhanced effect can improve the excitation efficiency of conjugated photosensitizers and promote the formation of triplet energy levels and the generation of reactive oxygen species (ROS), thereby strengthening the therapeutic outcome [93,94]. Furthermore, the synergistic interaction between photothermal and photodynamic effects can further amplify cytotoxic responses and significantly boost antitumor efficacy [95].

In summary, relying on the tunable optical response, the penetration advantage in the NIR region, and the strong electromagnetic field enhancement effect derived from LSPR, AuNPs serve as an important nanoplatform integrating diagnosis and therapy, exhibiting broad application prospects in the fields of nanomedicine and precision medicine.

### 2.3. Biological Properties

With the development of nanomedicine, AuNPs have exhibited broad application prospects in tumor theranostics, molecular imaging, and drug delivery. However, their in vivo behavior is complex, involving multiple factors such as stability, biodistribution, immune response, and long-term safety [96]. Therefore, systematically elucidating the biological properties of AuNPs is of great significance for evaluating their potential for clinical translation.

#### 2.3.1. Stability in Biological Fluids

Owing to the high surface energy of nanoparticles, bare AuNPs are prone to aggregation in high ionic strength or complex physiological environments, leading to increased particle size, altered optical properties, and even functional inactivation [97]. Therefore, improving their colloidal stability is essential for in vivo applications. The stabilization of AuNPs is generally achieved via two mechanisms: electrostatic stabilization and steric stabilization [98]. Electrostatic stabilization relies on repulsive forces generated by surface charges to maintain particle dispersion [99]. However, in salt-rich or high-protein environments, charge screening effects can compromise this stability [100]. In contrast, steric stabilization involves the formation of a hydration layer via surface modification with polymers (e.g., PEG, proteins, polysaccharides), which prevents direct contact between adjacent particles [101,102]. Under physiological conditions, stabilization based solely on charge is often insufficient; thus, polymer coating or biomolecule modification has become an important strategy for enhancing stability during blood circulation [103]. Kalmodia et al. [88] demonstrated that gold nanoparticles exhibited negligible SPR peak shifts (<5 nm) across different media, indicating excellent colloidal stability (Figure 2D).

Furthermore, when AuNPs enter the bloodstream or other biological fluids, plasma proteins typically adsorb rapidly onto their surfaces and form the so-called “protein corona” [104]. This process is driven by noncovalent interactions including electrostatic forces, hydrophobic interactions, and van der Waals forces, and can be classified into “hard corona” and “soft corona” based on binding stability [105]. Formation of the protein corona alters the surface chemical properties, hydrodynamic size, and surface charge of nanoparticles, thereby affecting their biological behaviors in vivo [106]. Particularly in active targeting systems, adsorbed proteins may partially mask targeting ligands originally modified on AuNP surfaces (e.g., antibodies, peptides, or aptamers), thereby reducing specific binding to tumor cell receptors and decreasing targeting efficiency [107]. On the other hand, enrichment of certain proteins in the corona (such as immunoglobulins or complement proteins) may promote recognition by macrophages and accelerate nanoparticle clearance, shortening blood circulation time and reducing accumulation efficiency in tumor tissues [108]. Therefore, when designing AuNPs for biomedical applications, it is necessary to not only ensure colloidal stability but also regulate protein corona formation via rational surface engineering strategies (e.g., PEGylation or biomimetic interface modification) to preserve the targeting ability and in vivo stability of nanoparticles.

#### 2.3.2. Biodistribution and In Vivo Transport Behavior

Biodistribution is one of the core factors determining the therapeutic efficacy and potential toxicity of AuNPs. The in vivo distribution of AuNPs is comprehensively regulated by particle size, morphology, surface charge, and surface modification [109]. In general, smaller particles (<10 nm) are more readily cleared by the kidneys, whereas larger particles (>200 nm) tend to be taken up by the mononuclear phagocyte system (MPS) and mainly accumulate in the liver and spleen [110,111]. Biodistribution studies by Sakr et al. [89] revealed that radiolabeled AuNPs predominantly accumulated in the liver and spleen, with only minimal distribution to the blood, lungs, and other organs (Figure 2E). In addition, positively charged particles usually exhibit higher cellular uptake but may also trigger stronger cytotoxic responses [112]. Neutral or PEG-modified particles tend to prolong blood circulation time and enhance passive accumulation in tumor tissues via the enhanced permeability and retention (EPR) effect [113,114]. Morphology also influences hemodynamic behavior. For instance, nanorods may display distinct boundary-layer motion in blood, thereby altering their tissue distribution pattern [115]. Therefore, rational tuning of particle size and surface chemistry enables the optimization of tissue distribution and targeting efficiency to a certain extent.

#### 2.3.3. In Vivo Biocompatibility and Toxicity Mechanisms

**Figure 3:**
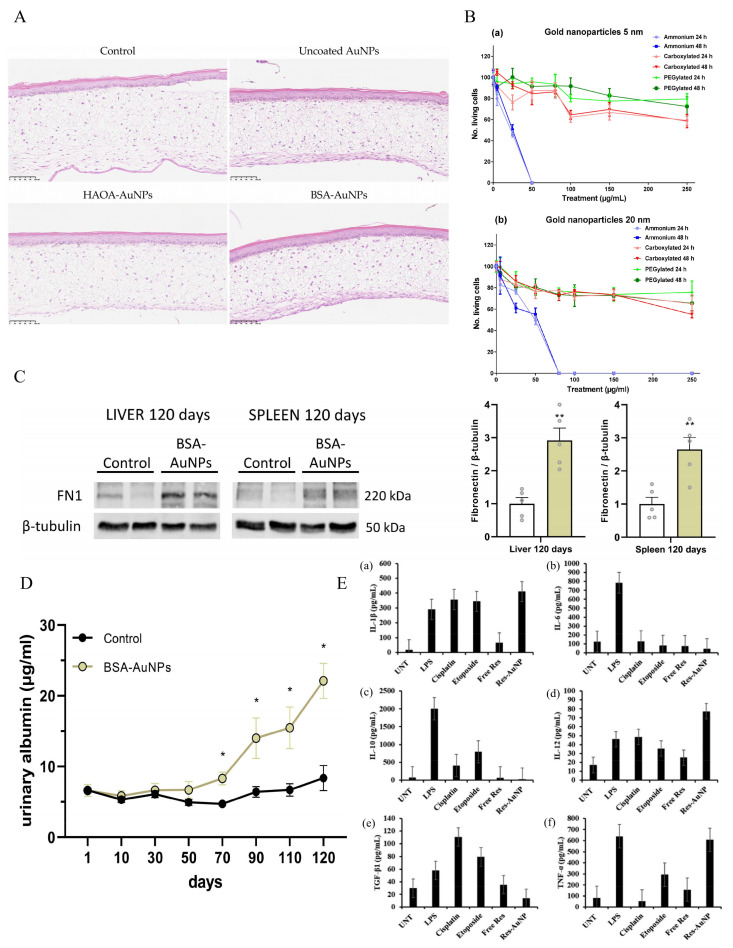
(**A**) Histopathological analysis (H&E staining) of skin models after incubation with different AuNP formulations. Reproduced from Ref. [116] under the terms of the Creative Commons license. (**B**) Cell viability of BEAS-2B cells after AuNP exposure at different sizes and time points. Reproduced from Ref. [123] under the terms of the Creative Commons license. (**C**) Fibronectin expression in treated and untreated groups after 120 days, assessed by Western blot. Data from Western blot represent the mean ± SEM of five independent experiments. Statistical significance among groups: *p* < 0.01 (**). Reproduced from Ref. [124] under the terms of the Creative Commons license. (**D**) Urinary albumin levels in nanoparticle-treated mice after 70 days compared with controls.Statistical significance among groups: *p* < 0.05 (*). Reproduced from Ref. [124] under the terms of the Creative Commons license. (**E**) Immunomodulatory effects of RESV–AuNP on RAW 264.7 macrophage cells compared with lipopolysaccharide (LPS), cisplatin, and etoposide treatment strategies. Each panel (a–f) represents the mean SD (n = 3) of key cytokine levels measured under different treatment conditions: IL-1β (**Ea**), IL-6 (**Eb**), IL-10 (**Ec**), TGF-β1 (**Ed**), IL-12 (**Ee**), and TNF-α (**Ef**). Reproduced from Ref. [89] under the terms of the Creative Commons license.

In vivo biocompatibility is an essential indicator for evaluating the clinical translation potential of AuNPs. Owing to the excellent chemical inertness of bulk gold, numerous studies have suggested that AuNPs possess favorable biocompatibility. Lopes et al. [116] demonstrated that AuNP formulations showed no significant morphological changes in a 3D human skin model, indicating good in vitro safety and biocompatibility (Figure 3A). However, at the nanoscale, their biosafety is influenced by multiple factors including particle size, morphology, dosage, surface modification, and administration route, warranting a more comprehensive and objective assessment of their in vivo behavior and potential toxicity [117,118,119]. The toxicity of AuNPs shows obvious size dependence. For example, smaller AuNPs may exhibit stronger cellular penetration, even entering the cell nucleus or crossing the blood–brain barrier, thereby increasing potential biosafety risks [120,121], whereas larger particles tend to accumulate in the liver and spleen over the long term. Such tissue distribution characteristics may result in prolonged retention of nanoparticles in local tissues and induce oxidative stress, inflammatory responses, or alterations in cellular function under certain conditions [122].

In addition, surface modification is regarded as a vital strategy for modulating the biocompatibility of AuNPs. Surface functionalization with molecules such as PEG, proteins, or polysaccharides can reduce immune recognition and phagocytosis to a certain extent, thereby prolonging blood circulation time and mitigating acute toxicity [125]. Vales et al. [123] reported that AuNP cytotoxicity is mainly determined by surface functionalization, with ammonium-modified NPs being most toxic, while carboxylated and PEGylated NPs show low toxicity (Figure 3B). Nevertheless, even after surface engineering, the long-term metabolism and clearance of AuNPs in vivo remain key issues of current research. Jakic et al. [124] reported that long-term exposure to Bovine serum albumin-coated AuNPs (BSA-AuNPs, 20 nm) led to increased fibronectin accumulation in the spleen and elevated urinary albumin levels, suggesting glomerular filtration impairment and potential chronic toxicity (Figure 3C,D). Different administration routes (e.g., intravenous injection, oral administration, or local delivery) also lead to distinct tissue distribution patterns, which further influence the overall toxicity evaluation [126].

Therefore, although numerous studies have demonstrated that rationally designed AuNPs exhibit favorable biocompatibility, their potential hepatosplenic accumulation, oxidative stress, and long-term retention cannot be neglected. Future research should integrate systematic toxicological studies, tissue distribution analysis, and mechanistic investigations of metabolic clearance to conduct a more meticulous and comprehensive assessment of the long-term safety of AuNPs, thereby providing a more reliable theoretical basis for their clinical translation.

#### 2.3.4. Immune Response and Inflammation Regulation

AuNPs can interact with the immune system, modulate macrophage activity and cytokine secretion, and complement system activation [127]. Several studies have indicated that unmodified or positively charged AuNPs are more likely to induce inflammatory responses, whereas neutral or hydrophilic surface modifications can reduce immune stimulation [128,129]. Within the TME, AuNPs may also regulate the infiltration and polarization of immune cells, offering potential strategies for combination immunotherapy [130]. Sakr et al. [89] showed that RESV (RESV; 3,5,4′-trihydroxy-trans-stilbene)–AuNPs modulate cytokine expression by downregulating IL-1β, IL-6, IL-10, and TGF-β1 while upregulating IL-12 and TNF-α, thereby promoting antitumor immune responses (Figure 3E).

In summary, the stabilization strategies, biodistribution profiles, and in vivo biocompatibility of AuNPs are interrelated and collectively determine their in vivo behavior and therapeutic efficacy. Rational design of particle size, optimized surface functionalization, and tailored interfacial properties can significantly improve the in vivo stability and safety of AuNPs, laying a solid foundation for their applications in tumor therapy, molecular imaging, and precision medicine.

#### 2.3.5. Effects of the TME on the Properties of AuNPs

The TME is recognized as a critical determinant governing the in vivo behavior and therapeutic response of nanomaterials. Its hallmarks include hypoxia, acidic pH, and an immunosuppressive state [131,132]. These characteristics not only influence the biodistribution and cellular uptake of AuNPs but can also further modulate their physicochemical properties and biological functions by altering interfacial chemistry and cell–material interactions [133]. Therefore, systematic consideration of the TME is of great importance when evaluating the in vivo performance and clinical translation potential of AuNPs.

First, the acidic TME (pH ≈ 6.5–6.8) exerts a significant influence on the surface chemistry and colloidal stability of AuNPs [134]. Under low-pH conditions, the ionization state of surface ligands or functional molecules may be altered, thereby modulating the surface charge density of nanoparticles and electrostatic interactions between particles [135,136]. Such changes may weaken interparticle repulsion and promote aggregation, which in turn affect their optical properties, biocompatibility, and cellular uptake efficiency. In addition, the acidic environment can induce conformational changes or bond cleavage in certain pH-responsive surface-modified materials, enabling controlled drug release or functional activation [137]. Therefore, interface design based on acidic responsiveness has become an important strategy for constructing tumor-specific AuNP nanosystems.

Second, the hypoxic microenvironment exerts indirect effects on the biological behavior of AuNPs by regulating the metabolic status and signaling pathways of tumor cells. Under hypoxic conditions, upregulation of hypoxia-inducible factors (HIFs) alters the expression of cell membrane receptors, endocytic pathways, and intracellular trafficking mechanisms, thereby influencing the uptake efficiency and intracellular distribution of nanoparticles [138]. Furthermore, in therapeutic strategies relying on ROS, such as PDT, insufficient oxygen availability limits ROS production efficiency and compromises therapeutic outcomes [139]. Consequently, recent studies have focused on introducing oxygen-generating components or designing hypoxia-responsive structures to improve the therapeutic performance of AuNPs in hypoxic TME [140].

Furthermore, the immunosuppressive TME plays a crucial role in regulating the in vivo fate of AuNPs. The accumulation of tumor-associated macrophages (TAMs), regulatory T cells, and various immunosuppressive cytokines in tumor tissues promotes the phagocytosis and clearance of nanoparticles, thereby impairing their accumulation efficiency and spatial distribution within tumor lesions [141]. Meanwhile, uptake of AuNPs by immune cells alters their effective local concentration in the microenvironment, which in turn influences imaging signal intensity or therapeutic efficacy [142]. On the other hand, AuNPs can also reversibly reshape the tumor immune microenvironment by modulating immune cell polarization and cytokine secretion, offering potential strategies for combination immunotherapy [143].

Collectively, the acidic, hypoxic, and immunosuppressive hallmarks of the TME exert multilevel effects on the physicochemical properties and biological functions of AuNPs by modulating their surface chemical status, aggregation behavior, cellular uptake, and in vivo distribution. Therefore, in the design and optimization of AuNPs, TME-related factors should be comprehensively integrated to enhance the performance of such nanoplatforms in diagnosis and therapy and further promote their clinical translation and application.

## 3. Applications of AuNPs in Cancer Detection and Diagnosis

Early diagnosis of cancer is critical for improving patient survival rates. However, conventional detection methods such as serological markers, imaging examinations, or tissue biopsy often suffer from insufficient sensitivity, high invasiveness, and poor real-time performance [144,145]. The development of nanotechnology has provided novel solutions for cancer detection. Among these, AuNPs exhibit unique advantages in molecular recognition and multimodal imaging due to their highly controllable size, morphology, and surface modification capability [146]. Table 2 provides a summary of representative applications of AuNPs in the detection of biomarkers and diagnostic imaging.

### 3.1. AuNP-Based Biomarker Detection Technologies

SERS represents one of the most representative applications of AuNPs in cancer detection. Through surface functionalization, AuNPs can be employed to construct high-sensitivity immunosensing platforms for the specific recognition of tumor markers including prostate-specific antigen (PSA) and alpha-fetoprotein (AFP) [147]. Zhong et al. developed a novel SERS-based acupuncture needles@gold nanoparticles@5-mercapto-2-nitrobenzoic acid (ANs@AuNPs@MNBA) probe, which enables simultaneous detection of pH and nitroreductase (NTR) in both solution and intact tissue samples, allowing in situ dynamic evaluation of the TME [148]. Furthermore, Mikhailets et al. utilized SERS enhanced by aggregated AuNPs to achieve sensitive and selective detection of pterins, particularly neopterin, in human serum [20]. Collectively, the integration of AuNPs and SERS enables ultrasensitive and highly selective detection of cancer-related biomarkers, applicable to both solution and tissue specimens, providing an effective strategy for early tumor diagnosis and precise assessment of the TME.

AuNPs are widely employed in electrochemical sensors for the highly sensitive detection of cancer biomarkers in body fluids, and can be integrated with microfluidic chips to achieve rapid, low-cost point-of-care testing (POCT). Tian et al. successfully developed a novel hierarchical nanocomposite TiO_2_-Ti_3_C_2_ MXenes@AgNPs, and established a dual-enhanced electron transfer ultrasensitive electrochemiluminescence (ECL) biosensing platform using three-dimensionally self-assembled AuNPs-ABEI nanoluminophores as catalytic amplifiers, which successfully detected the methylation of Septin9, a clinically relevant biomarker for colorectal cancer [150]. Liu et al. fabricated a sensing system using AuNPs for the in situ detection of microRNA-451 (miRNA-451), a key biomarker of colorectal cancer (CRC), achieving a limit of detection (LOD) of 19.2 fM [151]. Zhang et al. developed a self-assembled 3D/0D quasi-core–shell AuNPs@ZIF-90-COOH (Au@ZIF-C) for application in a fiber-optic biosensor for ultrasensitive detection of Helicobacter pylori [49]. The high surface area and functionalizability of AuNPs enable significant signal amplification in electrochemical sensors, allowing ultrasensitive, rapid, and low-cost detection of cancer and pathogen biomarkers.

### 3.2. Applications of AuNPs in Imaging Diagnosis

#### 3.2.1. Optical Imaging and NIR Imaging

AuNPs’ LSPR renders them high-contrast optical contrast agents. In particular, when the LSPR is tuned to the NIR region (700–1100 nm), optical imaging can be performed using NIR light with superior deep-tissue penetration, and combination with SERS further improves detection accuracy at the molecular level. Lu et al. reported a NIR-driven dynamic biosensing platform (JMMs-FNDs), which achieves dynamic capture and efficient enrichment of target molecules and probes [152]. Li et al. developed epigenetically modified DNAzyme-powered walkers (EMOWAs) immobilized on AuNPs for the simultaneous detection of O^6^-methylguanine-DNA methyltransferase (MGMT) and fat mass and obesity-associated protein (FTO) [153]. Overall, via LSPR modulation and functional modification, AuNPs enable technological expansion from deep-tissue optical imaging to multi-target molecular diagnosis, providing a critical platform support for precise cancer detection.

#### 3.2.2. PAI

PAI combines optical and ultrasound techniques, enabling imaging with greater depth and high resolution. AuNPs exhibit excellent photothermal conversion efficiency under NIR excitation and can serve as contrast agents in PAI to significantly improve the delineation of tumor boundaries, making them effective tools for the diagnosis of small lesions. Zhang et al. developed a novel tumor-targeted dual-modal nanoprobe (FFA), which exhibited outstanding bioluminescent and PAI performance in early tumor diagnosis [154]. Kim et al. introduced gold nanochain spheres (GSC) as high-efficiency PAI contrast agents, which generate stronger photoacoustic signals than conventional gold-based contrast agents [155]. Thomas et al. reported water-soluble NHC-stabilized AuNPs (NHC@AuNPs) synthesized via the TD method, which show distinct advantages in PAI [156]. In conclusion, through structural design and optimized surface functionalization, the signal enhancement capability and biocompatibility of AuNPs in PAI have been continuously improved, providing important technical support for precise tumor diagnosis and early screening.

#### 3.2.3. Multimodal Imaging

Multimodal imaging refers to the integration of two or more imaging modalities within a single diagnostic platform. In recent years, nanomaterial-based multimodal imaging platforms have become a research hotspot. Luo et al. synthesized alkaline phosphatase (ALP)-targeted, gadolinium-labeled AuNPs, designed for simultaneous detection by magnetic resonance imaging (MRI), computed tomography (CT), and fluorescence microscopy [157]. Lin et al. synthesized ND@Au, a core–shell nanoparticle consisting of a nanodiamond (ND) core doped with silicon vacancies (SiV) and a gold shell, which can serve as a potential imaging agent for Raman mapping, fluorescence imaging, two-photon fluorescence lifetime imaging (TP-FLIM), and high-resolution hard-X-ray microscopy in biological systems [158]. Overall, through functional modification and optimized structural design, AuNPs provide important technical support for the construction of high-sensitivity, high-resolution multimodal imaging platforms.

In summary, AuNPs show great scientific and clinical translational value in cancer detection and diagnosis, covering ultrasensitive molecular detection, deep-tissue imaging, and the construction of multimodal imaging platforms. With the collaborative advancement of nanomaterial engineering, artificial intelligence (AI), and clinical validation, AuNP-based cancer diagnostic strategies are expected to play an increasingly important role in precision medicine and early diagnosis.

## 4. Applications of AuNPs in Cancer Therapy

AuNPs exhibit remarkable potential in diverse cancer therapeutic strategies by virtue of their unique plasmonic optical properties, versatile surface engineering capability, and favorable biocompatibility. Recent studies have not only systematically advanced the applications of AuNPs in PTT and PDT, but also explored their multimodal synergistic therapeutic paradigms in radiosensitization, targeted drug delivery, and immunotherapy. Table 3 provides representative examples of AuNP-based applications in cancer therapy.

### 4.1. PTT and PDT

First, PTT is one of the most representative applications of AuNPs. This strategy offers advantages including strong spatial selectivity, minimal invasiveness, and low damage to surrounding normal tissues [175]. Numerous studies have demonstrated that the combination of PTT with other therapeutic modalities can significantly enhance antitumor efficacy. For example, in the in situ mineralized hydrogel (Fmoc-1-OH/AuNPs) based on self-assembled peptides constructed by Xu et al., AuNPs not only strengthened the photothermal effect but also enabled on-demand release of doxorubicin hydrochloride (DOX·HCl), leading to a markedly superior therapeutic outcome compared with monochemotherapy [159]. Mu et al. developed a biometallic peptide–drug conjugate (PGDC) composed of peptide nanofibers (PNFs), AuNPs, and DOX, which was incorporated into a photoresponsive double-network hyaluronic acid hydrogel to achieve synergistic photothermal/chemotherapy (PTT/CT) against breast cancer [160]. Chang et al. constructed a photothermal-promoted cascade nanozyme (TMnO_2_@Lap/PAH/L-Arg@Au) that further integrated photothermal, catalytic, and gas therapy functions, significantly inducing apoptosis and suppressing tumor growth [161]. In addition, AuNPs with different morphologies (e.g., nanorods, nanoshells, nanostars) exhibit distinct differences in light absorption and heat conversion efficiency, providing a structural basis for optimizing the performance of PTT [176].

Second, AuNPs can also serve as highly efficient enhancement platforms for PDT. By conjugating with photosensitizers or metal complexes, AuNPs can improve the generation efficiency of ROS and expand multimodal combination therapeutic strategies. For instance, Sliva et al. developed porphyrin-coated AuNPs (AuNPs@TMPyP) to integrate PDT with radiotherapy (RT) using photons and protons, which significantly enhanced radiosensitization and augmented therapeutic responses [162]. Bag et al. reported the conjugation of vanadyl(IV) complexes with AuNPs as promising metal-complex-functionalized gold nanoconjugates for PDT, offering a novel metal-based conjugation strategy for targeted cancer therapy [163].

Furthermore, synergistic therapy combining PTT and PDT is considered to possess broader application prospects. Haghighi et al. synthesized bilirubin–gold nanoconjugates (BGNCs), which exhibited excellent synergistic PTT/PDT anticancer efficacy in the HeLa cell model [164]. Xu et al. designed a dual-triggered NO-releasing nanomotor system with densely assembled AuNPs as the core and loaded with the NO donor N,N′-di-butyl-N,N′-dinitroso-1,4-phenylenediamine (BNN6), realizing the synergistic integration of gas therapy, PTT, and local hyperthermia to further enhance tumor suppression [165]. Overall, the multimodal optical therapeutic platforms constructed based on AuNPs achieve synergistic amplification of photothermal, photodynamic, and other therapeutic modalities through structural modulation and functional integration, providing an important developmental direction for precise cancer therapy.

### 4.2. Radiosensitization

AuNPs exhibit significant application potential in the field of radiosensitization. Owing to gold’s high atomic number (Z = 79), X-ray irradiation enhances the photoelectric effect and secondary electron emission, thereby improving local radiation dose deposition and ROS generation. This increases the sensitivity of tumor cells to radiotherapy while helping to reduce the overall radiation dose and minimize damage to normal tissues [177,178].

On this basis, AuNPs can further achieve multimodal synergistic sensitization through functional modification and drug loading. For example, Au-incorporated hyaluronic acid nanoparticles (Au/HAs) not only strengthen the radiosensitization effect but also induce immunogenic cell death (ICD) and activate antitumor immune responses [166]. AuNPs functionalized with RGD peptides and loaded with the histone deacetylase inhibitor SAHA show remarkable radiosensitization efficacy in non-small-cell lung cancer models [167]. In contrast, curcumin-coated AuNPs (Curc-GNPs) improve the therapeutic outcome of prostate cancer by combining chemotherapeutic and radiotherapeutic mechanisms [168]. Overall, AuNP-based radiosensitization platforms via structural design and functional integration provide an important developmental direction for achieving high-efficiency, low-toxicity precision radiotherapy.

### 4.3. Targeted Drug and Nucleic Acid Delivery

AuNPs present significant advantages in the delivery of anticancer drugs and nucleic acids. Through surface functionalization with antibodies, aptamers, or targeting peptides, AuNPs enable precise recognition and targeted delivery toward tumor cells, improving drug accumulation at tumor sites while reducing systemic toxicity. In chemotherapeutic drug delivery, a co-delivery system based on AuNPs and 5-FU exerted approximately 98% tumor growth inhibition in colorectal cancer peritoneal metastasis models via synergistic chemotherapy and PTT [169]. In gene therapy, PLK1 siRNA-loaded Au-PEI-PEG-AA nanoparticles combined with paclitaxel suppressed the NF-κB signaling pathway in prostate cancer, producing significant synergistic antitumor effects in xenograft models [170]. Furthermore, miRNA co-delivery systems involving AuNPs (e.g., miRNA-33a) markedly elevated the pro-apoptotic BAX/BCL2 ratio in breast cancer cells, further highlighting their potential in nucleic acid therapeutics [171]. Collectively, multifunctional delivery platforms constructed on the basis of AuNPs, through the synergistic integration of chemotherapy and gene therapy, provide a vital strategy for realizing high-efficiency, low-toxicity precision cancer treatment.

### 4.4. Immunotherapy Adjuvant Strategies

In recent years, the application of AuNPs in tumor immunotherapy has attracted increasing attention. AuNPs may not act directly as typical immunomodulatory agents, but indirectly influence immune responses by regulating the TME. They can promote the conversion of immunosuppressive TME toward an immune-supportive state, thereby enhancing the overall efficacy of cancer immunotherapy [179,180,181,182]. Studies have shown that by delivering tumor-associated antigens (TAAs), immunomodulatory antibodies, and gene-based drugs, AuNPs can reactivate the host immune response against tumor cells [183,184]. For example, Menka Khoobchandani et al. constructed mangiferin-functionalized AuNPs (MGF-AUCNPs) that modulate the immunological microenvironment of prostate cancer and significantly improve antitumor efficacy in mouse models [172]. Overall, the multifaceted roles of AuNPs in immune regulation and tumor immunotherapy provide an important theoretical basis and application prospects for the development of novel nano-immunotherapeutic strategies.

### 4.5. Multifunctional Nanoplatforms

**Figure 4:**
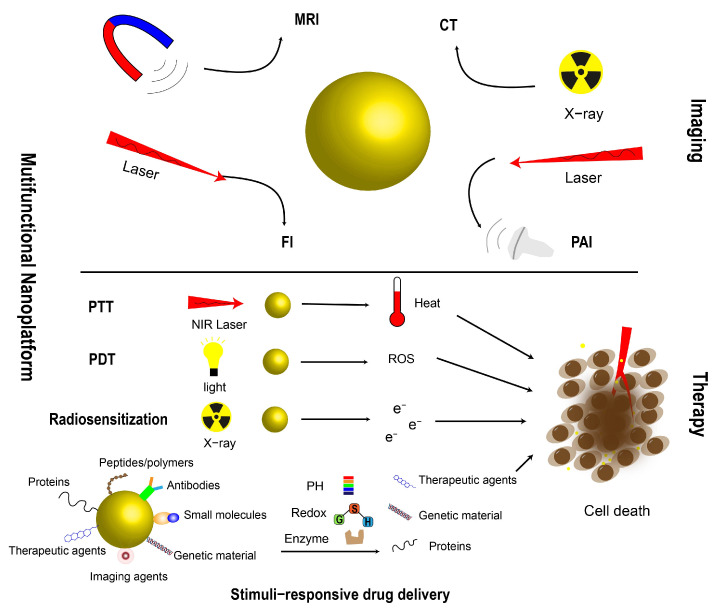
Schematic representation of gold nanoparticle (AuNP)−based multifunctional nanoplatforms for multimodal imaging, including magnetic resonance imaging (MRI), computed tomography (CT), fluorescence imaging (Fl), and photoacoustic imaging (PAI), as well as synergistic cancer therapy, including photothermal therapy (PTT), photodynamic therapy (PDT), radiosensitization, and stimuli−responsive drug delivery.

AuNPs can be integrated with chemotherapeutic agents, nucleic acid molecules, photosensitizers, and other components to construct theranostic systems that realize imaging-guided precise therapy. Moreover, through structural and surface functionalization design, AuNPs can be developed into intelligent, responsive, multifunctional nanoplatforms (Figure 4). In recent years, such platforms have gradually evolved toward stimuli-responsive modalities, including pH-, temperature-, or enzyme-responsive release mechanisms, to enhance tumor specificity and reduce systemic side effects. For example, the near-infrared-triggered DOX-PDA@Au-Au@PEG (DOX-PAAP) system enables controllable drug release in low-pH and high-glutathione (GSH) environments, thereby synergistically enhancing phototherapy and enzymatic therapy [173]. The MnO_2_-Au-BSA@CUR nanoplatform promotes the specific release of curcumin (CUR) in the acidic TME, improving tumor-targeted delivery efficiency and reducing systemic exposure [174]. Overall, intelligent multifunctional nanoplatforms based on AuNPs provide important technical support for precise cancer theranostics. However, their long-term in vivo safety, metabolic fate, and clinical translation feasibility still require further in-depth investigation.

In summary, AuNPs exhibit remarkable antitumor potential in PTT, PDT, radiosensitization, drug and gene delivery, and immune regulation due to their excellent optical properties and favorable functionalizability. Through multimodal synergy and intelligent responsive design, AuNPs are expected to achieve high-efficiency, low-toxicity precision therapy. However, further research is still required regarding their long-term safety and clinical translation.

## 5. Clinical Translation and Regulatory Considerations

### 5.1. Current Status of AuNPs in Clinical Trials

In recent years, AuNPs have attracted extensive attention in cancer diagnosis and therapy owing to their unique optical properties, favorable biocompatibility, and ease of surface functionalization. With the rapid advancement of nanomedicine, the applications of AuNPs in cancer theranostics have gradually progressed from basic and preclinical research toward clinical trials. Currently, a small number of AuNP-based systems have entered early-stage clinical trials, predominantly focusing on cancer therapy.

For example, CYT-6091, a tumor-targeting nanomedicine developed by CytImmune Sciences, represents one of the earliest AuNP-based formulations to enter human clinical trials. This platform achieves targeted drug delivery by conjugating tumor necrosis factor-α (TNF-α) onto the surface of AuNPs. In a phase I clinical trial involving patients with advanced solid tumors, it demonstrated favorable safety profiles and exhibited distinct tumor-targeting accumulation [185]. In addition, NU-0129, an AuNP–siRNA system structured as spherical nucleic acids (SNAs), has been investigated for gene therapy in glioblastoma, highlighting the potential of AuNPs in nucleic acid delivery and crossing the blood–brain barrier [186].

In addition to drug delivery systems, the clinical application of AuNPs in photothermal therapy has also garnered widespread attention. Clinical studies have explored the utility of AuNPs in PTT. For instance, a PTT system based on gold–silica nanoshells (GSNs), combined with magnetic resonance ultrasound fusion imaging, has enabled precise tumor localization and local thermal ablation in prostate cancer patients, demonstrating favorable safety and feasibility in preliminary investigations [187].

Notably, in addition to the aforementioned representative nanomedicine platforms, nanotechnology has also been employed for the modernization of traditional medical systems. For example, Nano Swarna Bhasma (NSB), a nanomedicine formulated by combining AuNPs with plant-derived bioactive components, has advanced from preclinical research to preliminary clinical application via green nanotechnology. In breast cancer models, this system exhibited dose-dependent tumor suppression, and favorable adjuvant therapeutic efficacy was observed in preliminary clinical studies of patients with metastatic breast cancer [188]. This study provides new insights into the application of AuNPs in the integration of traditional medicine and modern nanomedicine.

Overall, although the number of AuNP-based systems that have entered clinical trials remains limited, existing studies have preliminarily demonstrated their potential value in precision cancer diagnosis and therapy. To summarize the research progress of AuNPs in cancer theranostics, Table 4 lists representative preclinical and clinical studies, covering drug delivery, image-guided therapy, and multifunctional nanoplatforms. Nevertheless, AuNPs still face various challenges in the translational process from laboratory research to clinical application.

### 5.2. Lack of Sufficient Clinical Evidence

Despite the promising application potential of AuNPs in cancer theranostics, clinical evidence remains relatively limited, to date. Compared with nanomedicines that have achieved clinical translation, such as liposomes, far fewer AuNP-based systems have entered clinical trials, with the majority of studies still confined to in vitro experiments or animal models. Current clinical trials are mostly phase I studies, primarily focused on evaluating safety and tolerability, whereas large-scale clinical data supporting long-term efficacy, optimal dosing regimens, and therapeutic outcomes across different tumor types are lacking. In addition, the absence of standardized protocols for nanoparticle design, dosage, and evaluation criteria among different studies further complicates cross-study comparisons. Therefore, more well-designed, adequately powered clinical trials are warranted in the future to systematically assess the efficacy and safety of AuNPs and facilitate their clinical implementation in precision cancer therapy.

### 5.3. Long-Term Toxicity and Clearance Challenges

Long-term safety and in vivo metabolism represent major factors limiting the clinical translation of AuNPs. Unlike many degradable nanomaterials, gold is chemically inert and exhibits relatively slow metabolic clearance in vivo, which may lead to prolonged retention in organs of the reticuloendothelial system such as the liver and spleen [195]. Although numerous in vitro and animal studies have indicated that AuNPs possess low acute toxicity in the short term, their long-term biodistribution, metabolic pathways, and potential chronic toxicity remain insufficiently investigated. Moreover, the size, morphology, surface modification, and administration route of nanoparticles can all significantly influence their in vivo behavior. For instance, small-sized AuNPs are more readily excreted via the renal system, whereas larger particles tend to be taken up by the mononuclear phagocyte system and accumulate in organs [196]. Therefore, optimizing nanoparticle size, morphology, and surface functionalization strategies to facilitate in vivo clearance or enhance biodegradability is a key direction in current research [197].

### 5.4. Tumor Delivery Efficiency and Limitations of the EPR Effect

In addition to biosafety concerns, the in vivo delivery efficiency and tumor-targeting ability of AuNPs also represent critical barriers to their clinical translation. Most nanomedicines rely on the EPR effect for passive targeting, which arises mainly from abnormal tumor vascular architecture and impaired lymphatic drainage, enabling moderate passive accumulation of nanoparticles in tumor tissue [198,199]. However, a growing number of recent studies have indicated that the EPR effect exhibits substantial interindividual and tumor-type heterogeneity in human tumors, and its clinical consistency is far lower than that observed in animal models [200]. Furthermore, systematic analyses of various nanomedicines have revealed that typically less than 1% of systemically administered nanoparticles reach solid tumors in animal models, indicating persistently low overall delivery efficiency [201].

On the other hand, during systemic circulation, plasma proteins are prone to adsorb onto the surface of nanoparticles, forming a “protein corona”, which thereby alters their surface properties and in vivo behaviors and increases the probability of clearance by the mononuclear phagocyte system (MPS) [202,203]. In addition, the high heterogeneity of the TME, such as uneven vascular distribution, elevated tumor interstitial pressure, and dense extracellular matrix structure, may also significantly limit the deep penetration of nanoparticles in tumor tissues [204,205]. Therefore, relying solely on the EPR effect, it is often difficult to achieve stable and efficient tumor delivery. Future research needs to optimize nanoparticle size and surface modification, introduce active targeting strategies, and develop stimuli-responsive nanosystems to improve the accumulation and therapeutic effect of AuNPs in tumor tissues.

### 5.5. Comparison with Other Nanomaterials

When evaluating the clinical translation potential of AuNPs, it is of great significance to compare them with other common nanomaterial platforms. Currently, common nanopharmaceutical systems include liposomes, polymeric nanoparticles, and quantum dots. Compared with these materials, AuNPs possess unique LSPR properties, which endow them with significant advantages in fields such as PTT, PAI, and SERS detection. In addition, the surface of AuNPs can be easily subjected to various chemical modifications, enabling the construction of multifunctional integrated diagnostic and therapeutic platforms.

However, in terms of the maturity of clinical translation, other nanomaterials still have certain advantages. For example, a variety of liposomal nanomedicines have been approved by regulatory authorities and applied in clinical practice, with relatively mature pharmacokinetic characteristics and safety evaluation systems [206,207]. In contrast, uncertainties remain regarding the long-term in vivo retention and metabolic pathways of AuNPs. Meanwhile, polymeric nanoparticles generally have better biodegradability, while quantum dots exhibit outstanding performance in terms of fluorescence imaging sensitivity [208,209,210]. Therefore, different nanomaterials have their own advantages in terms of functional performance, biodegradability, and clinical feasibility.

To further compare the differences in clinical translation potential between AuNPs and other nanomaterial platforms, Table 5 summarizes the comparisons of several common nanomaterials in terms of advantages, limitations, and progress in clinical research.

### 5.6. Standardization Challenges in Nanomedicine Evaluation

The lack of unified standards for the nanomedicine evaluation system is also one of the important factors restricting the clinical translation of AuNPs. Unlike traditional small-molecule drugs, nanomaterials possess more complex structures and diverse physicochemical properties. Factors such as their particle size, morphology, surface charge, and surface modification methods may significantly affect their in vivo behavior. However, at present, there is still a lack of unified norms among different studies in terms of nanoparticle characterization methods, dosage standards, biosafety evaluation indicators, and pharmacokinetic analysis methods, which makes it difficult to directly compare the results of different studies. In addition, there are significant differences between in vitro experiments, animal models, and the human clinical environment, which also increases the uncertainty in the evaluation of the efficacy and safety of nanomedicines [211]. Therefore, establishing a unified and standardized nanomedicine evaluation system is of great significance for promoting the clinical translation of AuNPs in cancer diagnosis and therapy [212].

### 5.7. Good Manufacturing Practice (GMP) Production and Batch-to-Batch Reproducibility Issues

In the process of clinical translation of nanomedicines, large-scale production complying with GMP and batch-to-batch consistency are important factors affecting their industrialization [213,214]. Compared with traditional small-molecule drugs, AuNP-based systems usually have more complex structures, and their physical and chemical properties such as particle size, morphology, surface charge, and ligand density may significantly affect their in vivo behavior and therapeutic effects [109]. During the production process, even minor changes in process parameters—such as variations in reducing agent concentration, reaction temperature, or surface modification conditions—may lead to changes in the particle size distribution or surface properties of AuNPs, thereby affecting their biodistribution and therapeutic efficacy [215]. In addition, in the process of scaling up from laboratory scale to industrial scale, how to maintain the stability of nanostructures while meeting GMP standards is also a key challenge currently faced in the research and development of nanomedicines [216,217]. Therefore, establishing standardized production processes and strengthening the monitoring of critical quality attributes (CQAs) are of great significance for improving the reproducibility of the AuNP production process.

### 5.8. Regulatory Hurdles for Multifunctional Theranostic Nanoplatform

With the development of nanomedicine, multifunctional theranostic nanoplatforms have attracted widespread attention. Such systems usually integrate diagnostic, targeted delivery, and multimodal therapeutic functions, but their structural complexity also brings new challenges to regulatory approval [218,219]. Unlike traditional small-molecule drugs, multifunctional nanoplatforms are often composed of multiple functional modules, such as a nano-core, targeting ligands, therapeutic drugs, and imaging components, which makes their quality control and safety evaluation more complex. At present, regulatory authorities in various countries have not yet established a fully unified evaluation system for complex nanomedicines [220]. For example, in terms of product classification, such platforms may have the properties of drugs, medical devices, and diagnostic reagents simultaneously, leading to uncertainty in the regulatory pathway [221]. In addition, systematic research on release kinetics and interactions of different functional modules in vivo is still lacking, which also increases the difficulty of toxicological evaluation [222]. Therefore, establishing a more comprehensive regulatory framework for nanomedicines is crucial for promoting the clinical application of multifunctional nanoplatforms.

### 5.9. Conflicting Findings Regarding Toxicity, Biodistribution and Immune Responses

Although numerous studies have shown that AuNPs possess good biocompatibility under appropriate conditions, there are still certain discrepancies in the research results regarding their toxicity, biodistribution, and immunological effects [223]. In terms of toxicity, some studies have found that AuNPs modified with PEG or biomolecules exhibit low cytotoxicity at low doses [224], while other studies have indicated that AuNPs may induce oxidative stress and activate inflammation-related signaling pathways under high-dose conditions or with specific particle sizes [225]. In addition, small-sized AuNPs (<10 nm) are more likely to enter cells and even cell nuclei, which may have potential impacts on DNA stability. Regarding biodistribution, some studies suggest that ultra-small AuNPs (<5 nm) can be rapidly cleared through the renal system, while other studies have observed significant accumulation in reticuloendothelial system organs such as the liver and spleen [226]. Similarly, there are inconsistent conclusions on the effects of AuNPs on the immune system: some studies indicate low immunogenicity, while others have reported that AuNPs can induce the release of inflammatory factors or regulate immune cell functions [227]. Furthermore, differences in experimental models, administration doses, and characterization methods among different studies may further lead to inconsistencies in research results.

Overall, these seemingly contradictory research results are likely closely related to factors such as the particle size, morphology, surface chemical properties, dosage of AuNPs, and experimental models. Therefore, future studies need to systematically evaluate the long-term toxicity, biodistribution, and immunological effects of AuNPs under more standardized experimental conditions to more comprehensively understand their in vivo behavior and promote their safe clinical translation.

## 6. Conclusions and Future Perspectives

AuNPs possess unique physicochemical and biological properties, including tunable size and morphology, outstanding LSPR, and flexible surface functionalization. These characteristics endow AuNPs with distinct advantages in cancer detection, molecular imaging, and multimodal therapy. From ultrasensitive biomarker detection, NIR and photoacoustic imaging, to photothermal/photodynamic synergistic therapy, radiosensitization, targeted drug and nucleic acid delivery, and immune microenvironment regulation, AuNPs have gradually enabled the establishment of an integrated nanotheranostic system covering diagnosis–therapy–therapeutic monitoring. Through structural engineering and multifunctional integration, AuNPs not only significantly improve antitumor efficacy but also provide critical technical support for precision medicine and personalized therapy.

Despite the promising prospects of AuNPs in cancer theranostics, their clinical translation still faces certain challenges. Future research urgently needs to focus on improving clinical feasibility and safety. On the one hand, sustainable and scalable biosynthetic strategies should be developed, utilizing plants, fungi, and microorganisms to prepare AuNPs, thereby reducing environmental burden and enhancing biocompatibility. On the other hand, it is necessary to systematically evaluate their long-term toxicity, biodistribution, and metabolic fate, and establish a more comprehensive safety evaluation system to provide a reliable basis for clinical applications.

Meanwhile, the introduction of AI has provided new opportunities for the precise design of AuNPs and personalized medicine. AI-driven computational modeling can integrate patient-specific tumor biomarkers and TME characteristics to optimize the size, morphology, and surface functionalization of AuNPs, thereby achieving more targeted theranostic strategies. In addition, the further development of stimuli-responsive systems is expected to enable precise responses to intratumoral pH, enzymatic activity, or external physical stimuli, improving the spatiotemporal controllability of drug release.

Meanwhile, emphasis should also be placed on advancing the construction of integrated therapeutic platforms, which combine real-time multimodal imaging with stimuli-responsive drug release to achieve dynamic monitoring and precise regulation of the therapeutic process. Notably, the synergistic application of AuNPs with various therapeutic modalities, including PTT, PDT, radiotherapy, chemotherapy, and immunotherapy, has shown distinct advantages in overcoming tumor drug resistance and treating metastatic cancers. With the deep integration of materials engineering, AI, and clinical research, intelligent AuNP-based theranostic platforms are expected to provide novel solutions for personalized precision therapy against complex tumors.

## Figures and Tables

**Table 1:** Comparison of different gold nanoparticle (AuNP) morphologies and their corresponding therapeutic and imaging applications.

Morphology	Typical Size	Key Advantages	Therapeutic/Imaging Applications	References
Gold nanospheres	5–100 nm	Simple synthesis, high stability	Multimodal imaging-guided chemo-photothermal-chemodynamic synergistic therapy for TNBC; noninvasive photoacoustic imaging of cytotoxic T cell activity for monitoring immunotherapy response	[31,32]
Gold nanorods	10–100 nm	Tunable NIR plasmon resonance	Synergistic chemo-photothermal therapy and image-guided cancer theranostics; NIR-II fluorescence and photothermal signal-amplified lateral flow assay for ultrasensitive, multiplexed POC cancer diagnostics	[33,34]
Gold nanoshells	50–200 nm	Tunable optical absorption	Gold nanoshell-mediated NIR-triggered photothermal therapy combined with chemotherapy for enhanced tumor inhibition; electron-sink-enhanced SERS platform for real-time cancer cell detection and apoptosis monitoring via electron transfer	[35,36]
Gold nanocages	20–100 nm	High surface area, NIR absorption	Yolk-shell-satellite nanostructure-enabled SERS-LFIA for ultrasensitive and reliable detection of MMP-9 in disease diagnostics; FRET-regulated fluorescent nanoplatform for ultrasensitive detection of miRNA biomarkers in cancer diagnostics	[37,38]
Gold nanostars	20–150 nm	Strong electromagnetic enhancement	Molecularly imprinted polymer-assisted dual-mode SERS/colorimetric sensor for sensitive detection of CEA in cancer diagnostics; multiplexed SERS imaging for spatiotemporal profiling of CD8+ T cells and tumor biomarkers to predict immunotherapy response	[39,40]
Gold nanoprisms/nanoplates	20–200 nm	Strong plasmon resonance	Hotspot-engineered Au–Ag nanostructures for ultrasensitive SERS-based cancer biomarker detection; hybrid plasmonic nanocavity-enabled ECL biosensing for ultrasensitive extracellular vesicle detection and cancer metastasis diagnosis	[41,42]
Gold nanowires	nm–µm scale	High conductivity	Double gold nanowire-enabled plasmonic fiber biosensor for ultrasensitive refractive index sensing in cancer diagnostics; AuNP-stabilized nanowire-based electrochemical biosensor for ultrasensitive cytokine detection and cancer monitoring	[43,44]
Gold nanoclusters	<2 nm	Fluorescence, renal clearance	Antibody-directed nanotheranostics for targeted drug delivery, multimodal imaging, and immune modulation in inflammatory diseases; NIR fluorescence imaging-guided photothermal therapy with anti-metastatic effects and improved biocompatibility	[45,46]
Core–shell Au nanostructures	Variable	Multifunctionality	Electrochemical biosensing for ultrasensitive detection of miRNA biomarkers in cancer diagnostics; plasmonic biosensing for rapid and ultrasensitive detection of DNA methylation in early cancer diagnosis	[47,48]
Assembled Au nanostructures	Variable	Plasmon coupling	Au@ZIF-based plasmonic fiber biosensor for rapid and ultrasensitive detection of bacterial infection in clinical diagnostics; label-free electrochemical biosensing for sensitive detection of cancer biomarkers	[49,50]

Abbreviations: TNBC, triple-negative breast cancer; NIR, near-infrared; POC, point of care; SERS, surface-enhanced Raman spectroscopy; LFIA, lateral flow immunoassay; MMP-9, matrix metalloproteinase-9; FRET, fluorescence resonance energy transfer; CEA, carcinoembryonic antigen.

**Table 2:** Representative applications of gold nanoparticles (AuNPs) in biomarker detection and imaging diagnostics.

Strategy	Detection Principle	Target	LOD	Key Features	Reference
AuNP-enhanced SPR immunoassay	SPR with sandwich amplification	PSA	~10 ng/mL (direct); sub-ng/mL (with AuNPs)	Mixed SAM (16-MHA/11-MUOH) reduces non-specific adsorption; AuNPs enhance sensitivity; high-affinity cAbPSA-N7	[147]
ANs@AuNPs@MNBA SERS probe	Surface-enhanced Raman spectroscopy (ratiometric)	pH, NTR	pH: 4.75–7.25; NTR: 0–20 μg/mL	In situ, non-destructive tissue detection; acupuncture needle platform; dual-parameter sensing for tumor microenvironment	[148]
Aggregated AuNP-based SERS assay	Surface-enhanced Raman spectroscopy (intensity ratio analysis)	Pterins (neopterin)	16 nM	High sensitivity and selectivity in serum; characteristic Raman peaks (∼692, 1713 cm^−1^); ratiometric quantification using Amide I as internal standard; minimal sample preparation; rapid analysis	[149]
AuNPs-based ECL biosensor with dual-enhanced electron transfer	Electrochemiluminescence (dual amplification via SPR and electron transfer)	Methylated DNA (Septin9 gene)	8.2 aM	Ultra-high sensitivity; DNA tetrahedral probes improve binding efficiency; TiO_2_–Ti_3_C_2_ MXenes@AgNPs accelerate electron transfer; AuNPs-ABEI nanospheres enhance luminescence; validated in clinical serum samples with high accuracy	[150]
AuNP-based LSPR microfluidic biosensor	Localized surface plasmon resonance (dimerization-induced spectral shift)	miRNA-451 (CRC biomarker)	19.2 fM	Amplification-free detection; AuNP dimer formation induces LSPR red shift; single-particle tracking reduces spatial errors; multi-channel microfluidic chip enables in situ analysis	[151]
AuNPs@ZIF-90-COOH SPR optical fiber biosensor	Surface plasmon resonance (photoelectric enhancement)	*H. pylori*	1.37 CFU/mL	3D/0D quasi-core–shell structure enhances SPR response; high antibody loading capacity; rapid detection (16 s); applicable to gastric juice samples	[49]
AuNP-integrated NIR-driven dynamic fluorescence biosensor (JMMs-FNDs)	NIR-enhanced fluorescence detection with self-propelled nanomotors	miRNA-21, MUC1	0.72 fM	Self-propelled H-MnO_2_-Au nanomotors enhance mass transport; improved capture efficiency (≈52% → 78%); photostable FNDs reduce photobleaching; dynamic sensing outperforms static incubation (~40× sensitivity increase)	[152]
DNAzyme walker-based AuNP biosensor (EMOWAs@AuNPs)	DNAzyme activation + fluorescence amplification via walker motion	MGMT, FTO (demethylases)	8.8 pM (MGMT); 5.7 pM (FTO)	Autonomous DNA walker enables signal amplification (~100× improvement); epigenetic modification ensures high specificity; multiplex detection via Mg^2+^/Zn^2+^ orthogonality; applicable to cells and in vivo imaging	[153]
Folate-functionalized AuNR dual-modal nanoprobe (FFA)	Bioluminescence + photoacoustic imaging	Folate receptor-positive tumor cells	Not specified	Dual-modal imaging (bioluminescence + PA); AuNRs enable strong photoacoustic conversion; Fluc provides stable bioluminescence; folate-mediated active targeting; validated in vitro and in vivo with high biocompatibility	[154]
Gold nanosphere chains (GSCs) for photoacoustic imaging	Photoacoustic imaging (plasmonic coupling-enhanced photothermal conversion)	Tumor (targeted imaging)	Not specified	Chain structure enhances NIR absorption and photothermal conversion; stronger PA signal than AuNRs; high photostability; suitable for repeated imaging; validated in vitro and in vivo	[155]
NHC-stabilized AuNPs (TD-NPs) for photoacoustic imaging	Photoacoustic imaging (enhanced stability and photothermal response)	Tumor imaging (contrast agent)	Not specified	NHC ligands improve stability over thiol-based systems; top-down synthesized AuNPs show superior performance vs. bottom-up; excellent physiological stability; dual functionality (catalysis + imaging)	[156]
ALP-targeted Gd-labeled AuNPs multimodal imaging probe	MRI + CT + fluorescence imaging	ALP (liver cancer biomarker)	Not specified	Multimodal imaging (MRI/CT/Fl); ALP-targeted selective uptake; Gd(III) enhances MRI contrast; ~10× higher uptake in HepG2 vs. control cells; validated via cellular imaging and ultrastructural analysis	[157]
Nanodiamond@Au core–shell nanoparticles (ND@Au) for multimodal imaging	Raman + fluorescence + two-photon FLIM + X-ray imaging	Cells/in vivo models (zebrafish larvae)	Not specified	Multimodal imaging (Raman/Fl/TP-FLIM/X-ray); SiV-doped nanodiamond enables stable NIR fluorescence (~740 nm); Au shell provides optical and X-ray contrast; suitable for in vitro and in vivo imaging; potential for theranostics	[158]

Abbreviations: LOD, limit of detection; SPR, surface plasmon resonance; SAM, self-assembled monolayer; SERS, surface-enhanced Raman spectroscopy; NTR, nitroreductase; ECL, electrochemiluminescence; LSPR, localized surface plasmon resonance; NIR, near-infrared; FNDs, fluorescent nanodiamonds; MGMT, O6-methylguanine-DNA methyltransferase; FTO, fat mass and obesity-associated protein; PA, photoacoustic; NHC, N-heterocyclic carbene; MRI, magnetic resonance imaging; CT, computed tomography; Fl, fluorescence; TP-FLIM, two-photon fluorescence lifetime imaging.

**Table 3:** Representative AuNP-based nanoplatforms for cancer therapy and their major therapeutic outcomes.

Therapeutic Strategy	AuNP-Based Platform	Key Functional Components	Cancer Model	Main Outcomes	Reference
PTT + CT	Fmoc-1-OH/AuNPs	Fmoc-1-OH; AuNPs; DOX	In vitro and in vivo	Improved mechanical strength; laser-triggered reversible DOX release; enhanced PTT/CT synergy	[159]
PTT + CT	O-HAMA/PGDC	PNFs; AuNPs; DOX; O-HAMA	BC (in vitro/in vivo)	Rapid gelation; pH-responsive release; strong PTT; ~98% tumor inhibition	[160]
PTT + catalytic/gas therapy	MnO_2_@Lap/PAH/L-Arg@Au	MnO_2_; β-Lap; L-Arg; AuNPs	Tumor models	ROS-driven NO release; high PTT efficiency; mitochondrial apoptosis	[161]
PDT + RT/PTT	AuNPs@TMPyP	AuNPs; TMPyP; X-ray/proton	TNBC cells and spheroids	Enhanced ROS/^1^O_2_; radiosensitization; increased apoptosis	[162]
PDT	VO@AuNPs	Oxovanadium(IV) complexes; AuNPs	BC cells	Red-shifted absorption; enhanced ROS; light-induced apoptosis	[163]
PTT + PDT	Bilirubin–gold nanoconjugate (BGNC)	AuNPs; bilirubin	HeLa cells	High PTT efficiency; ROS generation; GSH depletion	[164]
Gas + PTT + SDT	Dual-triggered NO-releasing AuNP nanomotor	AuNPs; BNN6; NIR; US	Solid tumors	Enhanced penetration; ROS/RNS amplification; PA-guided therapy	[165]
RT + ICD	Au/HA NPs	Au/HA; SN38; CD44 targeting	Lung cancer (in vivo)	ICD induction; enhanced immune infiltration; abscopal effect	[166]
RT sensitization	RGD-AuNPs-SAHA	AuNPs; RGD; SAHA	NSCLC (A549)	Hypoxia suppression; enhanced radiosensitivity; apoptosis	[167]
RT sensitization	Curc-GNPs	AuNPs; curcumin	Prostate cancer (PC-3)	SER ↑ (1.82); ROS-mediated radiosensitization	[168]
NIR chemo–PTT	AuNP–5-FU	AuNPs; 5-FU	Colon cancer peritoneal metastasis	Tumor-localized hyperthermia; reduced HIPEC toxicity	[169]
siRNA delivery + CT	Au-PEI-PEG-AA/siRNA	AuNPs; PEI; PEG; anisamide	Prostate cancer	Efficient gene silencing; enhanced paclitaxel efficacy	[170]
Gene co-delivery	C-PEG-Nio-AuNPs	AuNPs; niosomes; miRNA	BC(MCF-7)	Lowest IC50; high apoptosis; BAX/BCL-2 ↑	[171]
Immunomodulation	MGF-AuNPs	AuNPs; mangiferin	Castration-resistant prostate cancer	M2 → M1 polarization; cytokine remodeling; tumor inhibition	[172]
PTT + enzyme	DOX-PDA@Au-Au@PEG	AuNPs; AuNCs; PDA; DOX	BC	Apoptosis–ferroptosis activation; ROS amplification	[173]
Hypoxia-relieved RT	MnO_2_-Au-BSA@CUR	MnO_2_; Au; BSA; CUR	BC (4T1)	O_2_ generation; enhanced radiosensitization	[174]

Abbreviations: PTT, photothermal therapy; CT, chemotherapy; AuNPs, gold nanoparticles; DOX, doxorubicin; BC, breast cancer; PNFs, peptide nanofibers; O-HAMA, oxidized methacrylated hyaluronic acid; ROS, reactive oxygen species; ^1^O_2_, singlet oxygen; PDT, photodynamic therapy; RT, radiotherapy; TNBC, triple-negative breast cancer; GSH, glutathione; RNS, reactive nitrogen species; SDT, sonodynamic therapy; NIR, near-infrared; US, ultrasound; PA, photoacoustic; HA, hyaluronic acid; ICD, immunogenic cell death; RGD, Arg–Gly–Asp; SAHA, suberoylanilide hydroxamic acid; NSCLC, non-small-cell lung cancer; SER, sensitization enhancement ratio; 5-FU, 5-fluorouracil; PDA, polydopamine; PEG, polyethylene glycol; PEI, polyethylenimine; BSA, bovine serum albumin; CUR, curcumin.

**Table 4:** Representative clinical and preclinical advances of gold nanoparticles (AuNPs) in cancer diagnosis and therapy.

AuNP Platform	AuNP Type	Cancer Model	Application	Study Type	Key Findings	Reference
CYT-6091 (TNF-α-AuNP)	PEGylated gold nanospheres	Advanced solid tumors	Targeted drug delivery	Clinical (phase I)	Demonstrated favorable safety profile and enhanced tumor accumulation	[185]
NU-0129 (SNA-siRNA AuN, P)	Spherical nucleic acids	Glioblastoma	Gene therapy	Clinical (phase 0)	Enabled efficient siRNA delivery across the blood–brain barrier in patients	[186]
Gold nanoshells (AuroShell)	Gold nanoshell	Prostate cancer	Photothermal therapy	Clinical study	Achieved precise image-guided tumor ablation with minimal off-target damage	[187]
Nano Swarna Bhasma (NSB)	Phytochemical-conjugated AuNPs	Breast cancer (preclinical models and patients)	Adjuvant nanomedicine therapy	Preclinical + Pilot clinical	Showed dose-dependent tumor inhibition and improved therapeutic outcomes in metastatic breast cancer patients	[188]
Ac-HSA-PLGA-AuNCs	Ultra-small AuNP clusters (<6 nm)	Tumor-bearing mice	Image-guided chemo-photothermal therapy	Preclinical study	Enabled efficient tumor ablation with improved renal clearance and reduced hepatic accumulation; demonstrated enhanced therapeutic efficacy and safety when combined with paclitaxel	[189]
Star-like polymer/AuNP/Temoporfin nanocomposite	Polymer-encapsulated AuNPs	Triple-negative breast cancer cells	Plasmon-enhanced photodynamic therapy	Preclinical study	Significantly enhanced ROS (singlet oxygen) generation via surface plasmon resonance, reduced dark toxicity, and improved intracellular delivery of photosensitizers, leading to increased tumor cell killing under low-power irradiation	[190]
Amine-PEGylated AuNPs (sphere/star/rod)	Gold nanospheres, nanostars, nanorods	Prostate cancer cells (PC3, DU145, LNCaP)	Radiotherapy sensitization	Preclinical study	Significantly enhanced radiosensitivity by increasing apoptosis and reducing cell viability; sensitization enhancement ratio (SER) was morphology- and cell line-dependent	[191]
Tf-functionalized AuPd BNP nanocomplex (DOX/5-FU)	Core–shell AuPd bimetallic nanoparticles	HeLa, MCF-7 (cancer) and HEK293 (normal) cells	Targeted dual-drug delivery	Preclinical study	High drug loading (>70%), pH-responsive release, transferrin-mediated targeting with selective cytotoxicity in cancer cells and minimal toxicity to normal cells	[192]
Multimodal gold nanostars (MGNs) with PET/SERS	Gold nanostars	CT26 and 4T1 murine tumor models	Multimodal immune imaging for immunotherapy response prediction	Preclinical study	Enables real-time in vivo tracking of CD8^+^ T cells and NK cells using combined immunoPET and Raman imaging, allowing early prediction of response to anti-PD-L1 + anti-CD47 therapy	[193]
KKS-Ru@AuNPs (RGD-modified Ru–Au nanoplatform)	Functionalized gold nanospheres	A549 lung cancer xenograft model	Targeted chemo-photothermal synergistic therapy with anti-metastasis effect	Preclinical study	Achieved enhanced tumor-targeting and synergistic therapy, resulting in 84.6% tumor weight reduction and significant inhibition of lung metastasis	[194]

**Table 5:** Comparison of representative nanomaterials: advantages, limitations, and clinical status.

Nanomaterial	Key Advantages	Main Limitations	Clinical Status
AuNPs	Strong optical properties; easy functionalization	Limited biodegradability; long-term accumulation	Early clinical trials
Liposomes	High biocompatibility; mature drug carriers	Stability and drug leakage issues	Several FDA-approved drugs
Polymeric NPs	Biodegradable; controlled drug release	Complex synthesis; possible immunogenicity	Under clinical investigation
Quantum dots	Bright fluorescence; stable imaging	Heavy-metal toxicity concerns	Mostly preclinical
Magnetic NPs	MRI contrast; magnetic targeting	Moderate sensitivity; safety concerns	Limited clinical use

Abbreviations: AuNPs, gold nanoparticles; NPs, nanoparticles; MRI, magnetic resonance imaging.

## Data Availability

No new data were created or analyzed in this study. Data sharing is not applicable to this article.

## Abbreviations

The following abbreviations are used in this manuscript:

TME	tumor microenvironment
AuNPs	gold nanoparticles
PTT	photothermal therapy
LSPR	localized surface plasmon resonance
NIR	near-infrared
PAI	photoacoustic imaging
PEG	polyethylene glycol
SERS	surface-enhanced Raman scattering
TEM	transmission electron microscopy
XRD	X-ray diffraction
PDT	photodynamic therapy
ROS	reactive oxygen species
EPR	enhanced permeability and retention
HIFs	hypoxia-inducible factors
TAMs	tumor-associated macrophages
PSA	prostate-specific antigen
AFP	alpha-fetoprotein
NTR	nitroreductase
POCT	point-of-care testing
ECL	electrochemiluminescence
CRC	colorectal cancer
LOD	limit of detection
MGMT	O^6^-methylguanine-DNA methyltransferase
FTO	fat mass and obesity-associated protein
MRI	magnetic resonance imaging
CT	computed tomography
AI	artificial intelligence
DOX·HCl	doxorubicin hydrochloride
PGDC	peptide–drug conjugate
PNFs	peptide nanofibers
RT	radiotherapy
BGNC	bilirubin–gold nanoconjugates
ICD	immunogenic cell death
Curc-GNPs	curcumin-coated gold nanoparticles
TAAs	tumor-associated antigens
MGF-AuCNPs	mangiferin-functionalized gold nanoparticles
CUR	curcumin
TNF-α	tumor necrosis factor-α
SNAs	spherical nucleic acids
GSNs	gold–silica nanoshells
MPS	mononuclear phagocyte system
GMP	good manufacturing practice
CQAs	critical quality attributes
